# SCAview: an Intuitive Visual Approach to the Integrative Analysis of Clinical Data in Spinocerebellar Ataxias

**DOI:** 10.1007/s12311-023-01546-0

**Published:** 2023-03-31

**Authors:** Mischa Uebachs, Philipp Wegner, Sebastian Schaaf, Simon Kugai, Heike Jacobi, Sheng-Han Kuo, Tetsuo Ashizawa, Juliane Fluck, EUROSCA study group, ESMI study group, RISCA study group, CRC-SCA study group, SCA Registry study group, Thomas Klockgether, Jennifer Faber

**Affiliations:** 1 Department of Neurology, University Hospital Bonn, Bonn, Germany; 2 DRK Kamillus Klinik, Asbach, Germany; 3 Fraunhofer Institute for Algorithms and Scientific Computing (SCAI), St. Augustin, Germany; 4 German Center for Neurodegenerative Diseases (DZNE), Bonn, Germany; 5 Bioinformatics Group, Department of Computer Science, Albert-Ludwigs-University Freiburg, Freiburg, Germany; 6 Institute of General Practice and Family Medicine, University Hospital Bonn, Bonn, Germany; 7 Department of Neurology, University Hospital Heidelberg, Heidelberg, Germany; 8 Department of Neurology, Columbia University, New York, NY, USA; 9 Department of Neurology, Houston Methodist Research Institute, Houston, TX, USA; 10 ZB Med, Information Centre for Life Sciences, Cologne, Germany; 11 Department of Geodesy and Geoinformation, University of Bonn, Bonn, Germany

**Keywords:** Spinocerebellar ataxia (SCA), Visualization, Observational studies

## Abstract

With SCAview, we present a prompt and comprehensive tool that enables scientists to browse large datasets of the most common spinocerebellar ataxias intuitively and without technical effort. Basic concept is a visualization of data, with a graphical handling and filtering to select and define subgroups and their comparison. Several plot types to visualize all data points resulting from the selected attributes are provided. The underlying synthetic cohort is based on clinical data from five different European and US longitudinal multicenter cohorts in spinocerebellar ataxia type 1, 2, 3, and 6 (SCA1, 2, 3, and 6) comprising > 1400 patients with overall > 5500 visits. First, we developed a common data model to integrate the clinical, demographic, and characterizing data of each source cohort. Second, the available datasets from each cohort were mapped onto the data model. Third, we created a synthetic cohort based on the cleaned dataset. With SCAview, we demonstrate the feasibility of mapping cohort data from different sources onto a common data model. The resulting browser-based visualization tool with a thoroughly graphical handling of the data offers researchers the unique possibility to visualize relationships and distributions of clinical data, to define subgroups and to further investigate them without any technical effort. Access to SCAview can be requested via the Ataxia Global Initiative and is free of charge.

## Introduction

In rare diseases, such as spinocerebellar ataxias (SCA), the number of patients at single centers is usually very limited. Therefore, the integration of clinical cohort data from different sources is desirable to create sufficient datasets to address scientific questions. For the most common spinocerebellar ataxias, SCA1, 2, 3, and 6, several longitudinal clinical cohort studies were successfully initiated and provided insights into the course of these rare disorders [1–13]. However, access to this cohort data is limited for various reasons. Not only data protection poses a challenge, but also inhomogeneity of the data, which often requires extensive, largely manual pre-processing. Unification, normalization, and re-coding of the data is necessary for the integrative analysis due to inconsistent storage and coding of data from different sources. Since such data processing and unification is challenging and time-consuming, in practice, this task is not carried out by the clinical expert but by a data analyst who is lacking the knowledge of the clinical details. As a result, there is often a significant delay and communication gap between data analyst and medical scientist, which (1) in the worst case leads to a loss of information due to misattribution of analogies and (2) imposes considerable limitations on the options for hypothesis-free data evaluation. Accordingly, in our project, clinical scientists and technical experts were involved. We defined a model to specify analogies and transformations of heterogeneous data sources based on a consented tabular format to ascertain a scientifically correct and technically unequivocal definition of analogies of the data to be pooled.

Moreover, the abovementioned communication gap impairs the analysis of scientific data, the generation and evaluation of hypotheses, and the quick and easy hypothesis-free visualization and browsing of existing data. In practice, the clinical scientist asks the data analyst to process and plot the data based on a predefined selection of hypotheses. Ideally, additional hypotheses from the visual inspection of the resulting plots can be derived, and subsequently, the clinical scientist refines his requests to the data analyst. Data visualizations that would be accessible without much technical effort, such as direct graphical handling of data, would provide researchers with the unique opportunity to explore underlying relationships of clinical, demographic, and genetic data themselves. It would allow clinical scientists to browse data in a hypothesis-free manner and gain a better understanding of relationships, subsets, and limitations independently and immediately.

With “SCAview,” we introduce an easily accessible online tool that allows to visualize, select, and compare clinical data of SCA1, SCA2, SCA3, and SCA6 mutation carriers. The graphical handling enables users to easily visualize distributions of and relations between clinical attributes. For SCAview, we integrated SCA research cohort data consisting of up to now 1417 mutation carriers with 5509 clinical visits over an observation period of up to 8 years from 5 different European and US cohort data sources [1–13]. In the open release, we implemented a synthetic version of the integrated data. SCAview is freely available for all registered members of the Ataxia Global Initiative (AGI) and for interested researches upon reasonable request.

## Methods

### Integration of Data

The basis for the integration of data from five SCA cohorts, namely ESMI, EUROSCA, RISCA, SCA Registry, and CRC-SCA, was the precise definition of analogous and comparable variables as well as of the encoding used for defined values in each data source. First, we defined a data model for every attribute included in SCAview, like sex, and age of onset, ataxia severity scale values. Second, we mapped or transformed equivalent, analogous, and comparable attributes of each source data set. Mapping and transformation of source data to a common data model ranges from simple cases to more complex transformations that require precise knowledge of the study design and data. Trivial is the mapping of different identifiers for unequivocal attributes, like “DateOfBirth,” “yob,” or “DOB” for the date of birth. In other cases, simple calculations can be applied, e.g., for age of onset versus year of onset in combination with year of birth. Other attributes require precise knowledge about the design, e.g., in the case of age of onset (attribute “aoo”). In the source data of SCA Registry, ESMI, EUROSCA, and RISCA age of onset is defined as the reported first occurrence of gait disturbances. Consequently, it needs to be mapped onto “WALKINGPROBLEMSYEARS” of CRC-SCA data. Finally, e.g., for the transformation of disease stages one has to agree on a consensus of transformations. Representative examples of the underlying mappings and transformations can be found in Suppl. Table 1 as well as in the documentation section of the viewer. For each attribute included in SCAview, the distinct mapping, re-coding, and transformation steps that need to be applied to the source data was determined by clinical scientists in a comprehensive textual format in a tabular sheet. Third, we developed an import protocol, that reads the source data, provided in the frequently used CSV or EXCEL format, unifies the data based on the defined mappings, and reformats the data to an EAV-format (Entity–Attribute–Value) that represents one data point per line (e.g., Entity: PatientXYZ at time-point 2006_09_22, Attribute: SARA_GAIT, Value: 3). This data-format allows the simple concatenation of increasing amounts of data. Since the timestamp of one study visit can be spread over a certain range, even more when clinical routine data is used to enlarge the database, we extended every entity by a distinct value for the visit. We clustered timestamps within a range of < 28 days as a cluster or in other words as one visit. This definition is a given pre-setting during the import process. For non-dynamic information that is time-invariant like date of birth, genetic information or sex, the timestamp and visit information is not valid and the respective attributes are marked as static in the data model.

Once defined, the mapping or more concretely the import can be applied repetitively if the source data is enlarged without any additional effort as long as the data model does not change. Finally, the import protocol performs data sanitizing by checking for plausible values (data types and ranges).

Attributes included in SCAview are demographic information (age, sex), characterizing data (such as CAG repeat length of the longer and shorter allele, age of onset if applicable) and visit-related assessments of disease severity: Scale for the Assessment and Rating of Ataxia (SARA, 8 sub-items and sum score) [14], Inventory of Non-Ataxia Signs (INAS, categories and count) [15], and disease stage [16]. The current available attributes are listed in the Suppl. Table 1. Moreover, a complete list is deposited in the viewer as well.

### Creation of the Synthetic Cohort

In order to prevent the traceability of participants and since SCAview employs partially unpublished data, the final integrated SCAview dataset was fed into a noise-adding model for the variables age, CAG repeat length of the longer as well as shorter allele, and the reported age of onset to generate a synthetic cohort. The conceptual and technical details are described in Wegner et al. (https://doi.org/10.48550/arxiv.2210.16649) [17]. The synthetic cohort preserves basic correlations and distribution properties. To the timestamp of the date of birth, we added a random number of days resulting in a random value within the range of ± 1.5 years. Notably, timestamps that would allow to identify visit or birth dates are not included in the viewer. A discrete synthetic cohort’s variable $\tilde{X}$ is synthesized from a variable *X* of the original SCAview dataset via an affine noise-adding model $\tilde{X}=f(X)=X+\in$, where ϵ is randomly drawn from a normal distribution located at zero with a variance corresponding to the variance *Var*(*X*) of the original variable *X* scaled by a parameter *b*, that was optimized for each variable separately with respect to its distribution. The model preserves a variable’s mean ($E[\tilde{X}]=E[X]$) as well as the pair-wise Pearson correlation coefficient up to a scaling factor: $\rho_{\tilde{X},\tilde{Y}}=\frac{1}{1+b}\rho_{X,Y}$. This model was preferred over other approaches due to its simplicity and computational efficiency that in addition allows a convenient application to other cohorts perspectively (for more details see: https://doi.org/10.48550/arxiv.2210.16649) [17]. In addition, the original cohort pseudonyms have been replaced and the random assignment to the new identifier is not stored. In order to achieve GDPR compliance while maintaining the utility of data, these steps were implemented and, together with the high effort for filtering at the individual level, considerably minimize the possibility of re-identifying an individual.

### Browser-Based Visualization Tool

To enable the clinical scientist to browse cohort data, we developed an intuitive tool for easy data visualization. Existing tools for data handling and visualization beginning from simple spreadsheets like Microsoft’s Excel (www.microsoft.com) to mathematical analysis tools like Graphpad Prism (graphpad.com) and programmable data analysis software like IGOR (www.wavemetrics.com) as well as IBM’s SPSS, SAS, GraphPad Prism, TIBCO Spotfire, and R. The most prominent example for more medical informatics oriented platform is i2b2 (www.i2b2.org), designed for professional data organization and field-specific data science approaches. However, apart from necessary data annotation steps, those applications require either time-consuming manual sorting, selection, and pairing of data to plot it for every single request or, if they are more powerful and provide some form of automation, they presume an advanced technical expertise and pre-customization for use. Therefore, they are often primarily handled by non-clinical experts like statisticians. In all tools, we discovered the user has to perform data handling in a tabular and numeric manner first, gaining a visualization in subsequent steps. Consequently, such solutions lack a first-glance visual exploration of the data. To overcome this limitations, SCAview is designed as a web-based application requiring no installation or setup. SCAview constructs from a data service that works as a backend module for processing and providing the data, and a visualization service, that works as a frontend the user interacts with. The backend module comprises a Django (https://www.djangoproject.com/) application that handles the data logic and a Redis DB (https://redis.io/) enabling quick data access. The frontend is developed using React (https://reactjs.org/) and the React version of Plotly (https://plotly.com/javascript/react/) to create interactive plots.

## Results

In the final data set, we included 1417 subjects (264 SCA1, 297 SCA2, 551 SCA3, 206 SCA6, and 99 healthy controls (HC)). Thirty-one subjects were excluded due to missing or invalid genetic information, 16 subjects were excluded due to invalid SARA item scores (× 0.5-scoring in items that only allow for integer values, namely gait, stance, sitting, and speech) and 5 subjects for which only characterizing and demographic but no visit-specific data were available. Since in the field of ataxia research some consented scales and assessments are established (SARA score, INAS count), the data was equivalent in many cases. In other cases, the data describes similar aspects but uses different scales and marks to define. Following the mapping or transformation and merging of the data, we evaluated the homogeneity of the different sources by a first visual inspection revealing no obvious differences. Nevertheless, this paper does not address the question of statistical homogeneity or potential differences of the different cohorts. Except for missing data in single subjects, all attributes included in the actual version of SCAview (see Suppl. Table 1) were represented in all sources, except for the disease stage in the RISCA cohort of at-risk subjects of SCA1, 2, 3, or 6 mutation carriers [4, 6]. All 1417 accepted subjects form the basis to create the synthetic cohort. This synthetic or virtual cohort is the data set that can be explored within SCAview.

### Data Browsing

SCAview was conceptualized for the first-glance visualization of datasets and graphical handling of all data processing. Consequently, the browsing of data is based on the graphical presentation of selected attributes from the beginning. The current version provides four plot types: (1) histograms and (2) scatter plots to depict the distribution and correlation of numerical data, respectively as well as (3) bar graphs to quantify categorical attributes. Finally, (4) timeline plots to visualize the development of attributes over time. In the latter plot type, the value of each data point is depicted versus its timestamp in relation to a reference date (e.g., date of birth or disease onset). The resulting visualization is then, e.g., the SARA sum score plotted against age or SARA sum score plotted against disease duration.

**Fig. 1:**
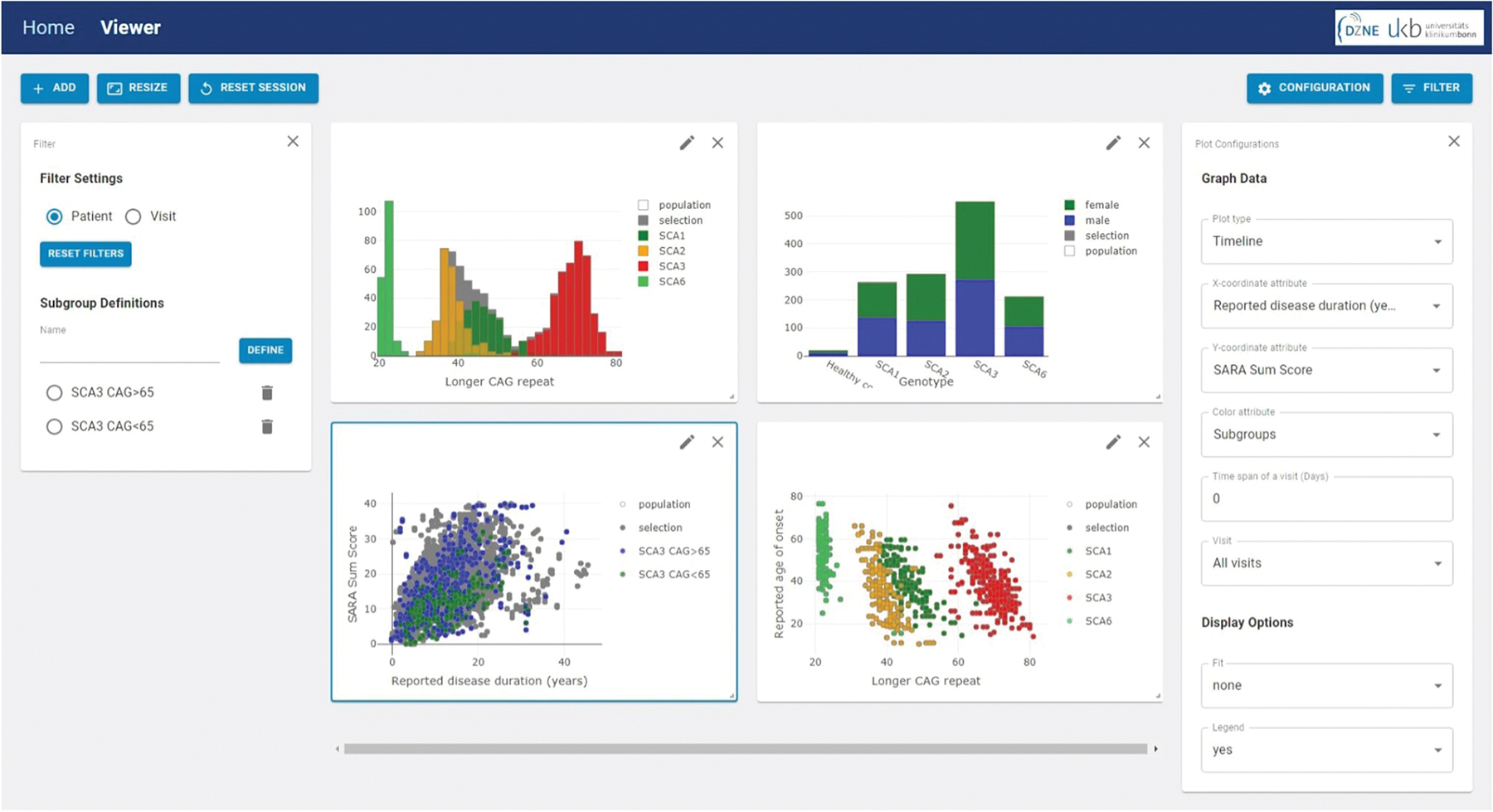
Example Screenshot of SCAview. On the left side one can see the filter-setting window, in the middle section, four examples for the different plot types available are depicted and on the right side the configuration window for the actual plot is displayed (here left bottom scatter plot, marked with a blue outline). The plot in the upper left corner shows a histogram of the CAG length of the longer allele with the genotype as additional color-coded attribute. The right upper corner shows a bar plot of each genotype with sex as color-coded additional attribute. In the left bottom corner, a timeline plot of SARA sum score versus reported disease duration displays two subgroups that were previously defined by filter settings: SCA3 mutation carriers with a CAG repeat length of more and those with less than 65 CAGs of the longer allele. The example plot on the right bottom corner shows a scatter plot of the reported age of onset versus CAG repeats on the longer allele, again with the genotype as additional color-coded attribute

In all types of plots, an additional attribute can be selected to color the data points. If a categorical attribute is selected, it is represented by different distinct colors. Numerical values spanning across a particular range, e.g., SARA sum score or CAG repeats, are encoded according to a color map. Multiple plots can be open in parallel. Figure 1 shows example screenshots of the SCAview user interface.

### Filtering

**Fig. 2:**
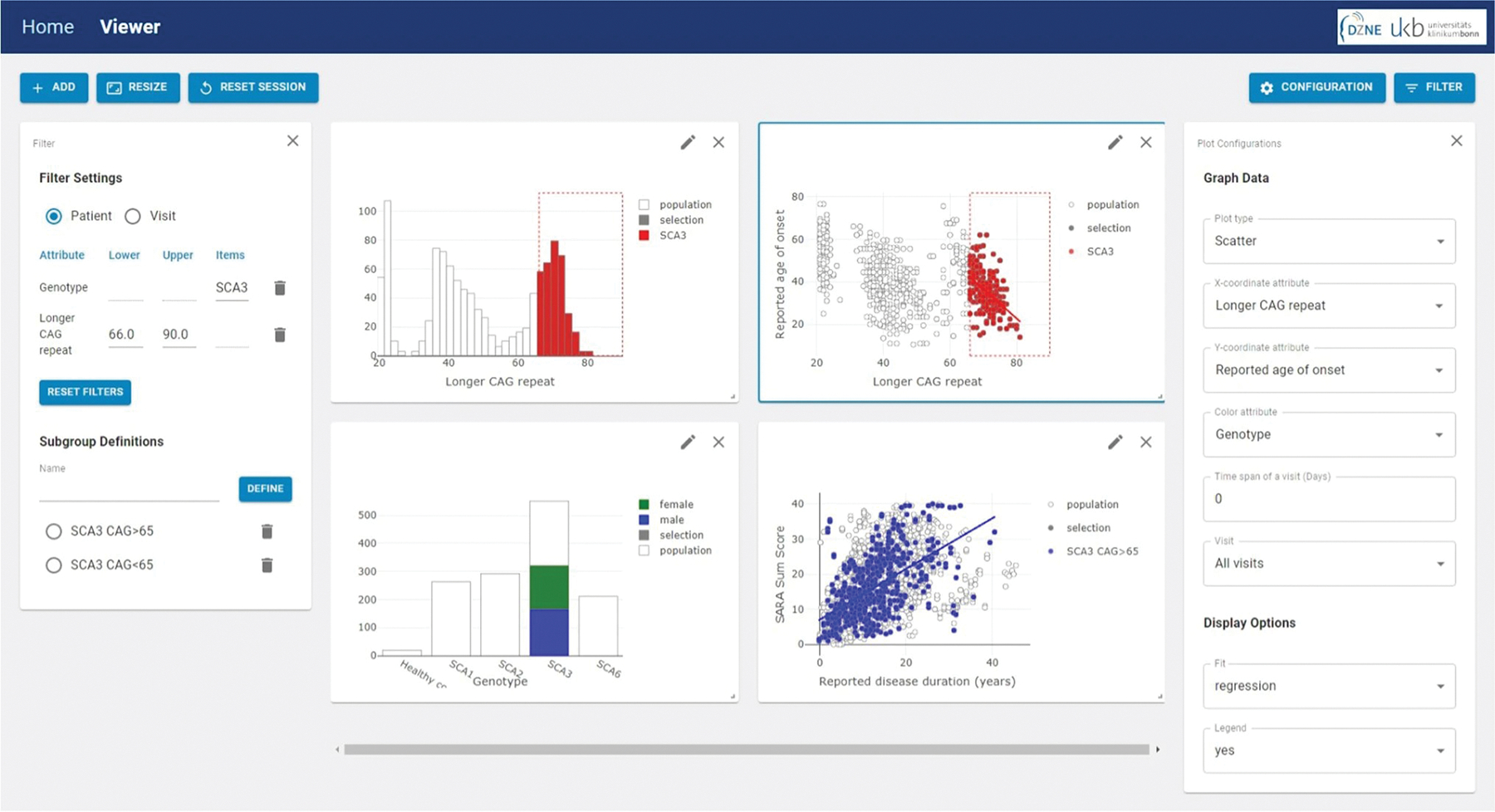
Shown here is an example screenshot of plot types in SCAview with the applied graphically defined filtering of (i) SCA3 (box selection in the bottom left plot) + (ii) CAG > 65 (box selection in the upper left plot). The bar and timeline plots of the bottom row are the same as in Fig. 1 (right upper corner and left bottom corner). Notably, now only the selected subcohort defined by filter settings of “SCA3 + CAG > 65” is depicted with the entire population shadowed in the background

In the beginning, the complete data set is depicted. According to the graphical handling of data processing, subgroups can be defined via a selection tool in any graph. For example, if a clinical scientist is only interested in SCA3 mutation carriers with more than 65 CAGs in the longer allele, one can easily select the SCA3 in the bar plot of genotype and the CAG repeat range in the histogram of longer allele CAGs, respectively. The chosen filters are automatically applied to the dataset, and in all generated plots, the selected subgroup is highlighted, while the excluded data is grayed out (Fig. 2). This cross-filtering allows one to define a filter in one graph and observe the effect of the selection in all other visualizations. After choosing a selection defined by particular filters, one additional filtering option has to be set. Since for most patients in the dataset multiple values for a specific attribute have been measured over the time of observation, two different filter concepts in addition to the chosen selection characteristics are provided: (i) patient-wise filtering and (ii) visit-wise filtering. In the setting of patient-wise filtering, subcohorts include all patients who have at least one visit with the specific subcohort-defining attribute. In contrast, the visit-wise filtering does not select patients, but only those visits, where the attribute(s) fits to the subcohort definition. One concrete example to outline the differences in the patient-vs. visit-wise filtering would be as follows: The researcher is interested in subjects with a SARA sum score of > 20. Subject no. 100 has 3 visits, with a SARA sum score of 12, 16, and 22, respectively. In the patient-wise setting, subject no. 100 would be included in the selection “SARA sum score > 20” with all three visits. In contrast, in the visit-wise setting, subject no 100 would be included in the selection “SARA sum score > 20” only with its last visit.

### Subgroups

*A*ll applied filters are summed up in a tabular representation. Here, a specific filter set can be defined and labeled as a subgroup. These subgroups act as bookmarks to recall this set of filters. In addition, defined subgroups can be used to group the data in a plot to perform a direct visual comparison of two or more selected subgroups (see timeline plot of disease duration versus SARA sum score in the left lower corner in Fig. 1, comparing SCA3 mutation carriers with more than 65 CAG repeats in the longer allele compared to those with less).

## Discussion

### Integrated Data set

Spinocerebellar ataxias (SCAs) are the most common autosomal-dominantly inherited ataxias worldwide and have been studied in several European and US longitudinal observational studies. We had access to longitudinal cohort data from EUROSCA, RISCA, ESMI & SCA Registry, and CRC-SCA that comprise together more than 5500 visits of more than 1400 SCA1, SCA2, SCA3, and SCA6 mutation carriers. The integrated data formed the basis to create a synthetic cohort for SCAview. Since it is the first integration of the most extensive cohort studies available, it is unique in its size. To enable a wide scientific community of ataxia researchers to browse and explore this integrated dataset in a first glance in SCAview, we had to shuffle the data due to data protection reasons and of partly unpublished data. Correlations and distributions within the created synthetic cohort are largely kept [17]. To ensure that no subject can be re-identified even with the creation of this synthetic version of a virtual cohort we did not include the study site in SCAview. To include at least the continent of origin might be possible and still maintain data protection valid as more data sets become available.

### Facilitated Data Inspection

Inspection of such extensive data, the selection of subgroups according to specific features, the visualization of correlations, and subsequent generation of hypotheses and their evaluation are common procedures in clinical science. However, processing, selection, and visualization of the data requires technical expertise, is time-consuming, and therefore frequently carried out by a data scientist lacking the clinical knowledge resulting in delay or even misunderstanding.

To enable the scientists and researchers to explore data without the service of any data scientist, the basic concept of SCAview is the intuitive first-glance visualization of datasets and graphical handling of all data processing. This contrasts with most existing tools, where the initial step is a hierarchical, textual, and logical definition of a cohort of interest. These tools include widely used spreadsheet applications like Microsoft’s Excel. Beyond, more powerful, scripting-oriented tools are, e.g., IBM’s SPSS, SAS, GraphPad Prism, and TIBCO Spotfire, however addressing primarily statisticians. The open-source software R might additionally be used by data scientists, who focus more on frameworks like Python and JavaScript, which, like R, provide a vast number of libraries for data handling, statistical tests, and visualizations. While all these solutions require education in programming, SCAview opens the data to an integrative, quick visual analysis, hypothesis generation, and evaluation in a synthetic cohort representative for the integrative dataset of large longitudinal observational cohorts in SCA1, SCA2, SCA3, and SCA6. In practice, the visualization of SCA data with SCAview presented here were tested and used to demonstrate distribution and relations of clinical data within the research contexts in the German Center of Neurodegenerative Diseases (DZNE), Bonn, in presentations as well as internal scientific reports and educational trainings. However, as outlined above SCAview is based on a synthetic cohort. SCAview explicitly does not provide options of statistical testing or display of correlation coefficients, as this might lead to the false assumption of real data. Consequently, we did not include any statistical testing to avoid misinterpretations. Moreover, we would like to point out that no conclusions on an individual level can be drawn, neither for prediction of diagnosis nor for clinical deterioration. SCAs show a high level of intraindividual variability even in mutation carriers of the same family. Initiatives like the Critical Path to Therapeutics for the Ataxias (CPTA, https://c-path.org/programs/cpta/) offer perspectives for researches to get direct access to real data of large ataxia cohorts. However, we are convinced that SCAview has an added values for ataxia researchers since it is unique in allowing scientists to become familiar with the relations of clinical data within SCA1, 2, 3, and 6 on their own without technical effort, increase knowledge, and to develop hypotheses.

### Perspectives

We consider SCAview as an active development and objective of future research. Any updates and further developments will be documented and made available in the documentation page. Our aim is to continuously extend the underlying datasets by including additional visits of ongoing longitudinal cohort studies and additional worldwide cohort data. Moreover, we aim to increase the number of available attributes. In particular, we aim to include patient-reported outcome measures. However, as outlined in the results section, this work does not address the question of the homogeneity of the different sources but provides a database for visualization. Before the integration of new cohort data, in particular from non-European and non-US sites, a data check to ensure consistency of typical features (e.g., such as progression rates) needs to be tested.

The challenge to overcome the communication gap between clinical and technical experts resulted in (1) a simple but ubiquitary available, comprehensive, and easily re-usable model to define data mapping or transformation and (2) an intuitive visualization tool with overall graphical handling. Perspectively, the benefit of an easy and intuitive handling of clinical datasets should be rendered available for individual local projects and datasets. Currently, the internal importer protocol is code-based and limited to the data downloads of the included cohorts. The usage of input data in form of tables in common formats is in preparation. Again, the aim is to keep the simplicity and independence from IT support for the clinical user. Like SCAview, such a future upload setting should be based on technologies that do not need any installation of databases or administrator rights with a user-friendly guidance, e.g., via graphical user interfaces (GUI). We do not envision to automatically integrate local data in the main SCAview data set. Local data will be visible for the uploading researcher only and can easily be identified via filtering and thereby color coded as a third item in any plot.

Taken together, SCAview presents a novel and unique insight into clinical aspects of the most common spinocerebellar ataxias, SCA1, SCA2, SCA3, and SCA6, by presenting the first unified and merged cohort data and making it accessible to a broad community of interested researchers and clinicians by providing an intuitive tool to explore and visualize these data.

## Supplementary Material

Supplementary1

## Data Availability

Access to SCAview (visualization of the synthetic cohort) is available for all members of the Ataxia Global Initiative (AGI) and can be granted upon reasonable request for academic non-AGI members. Availability of the data of observational studies has to be requested at the respective study PIs (Thomas Klockgether for ESMI and SCA Registry Tee Ashizawa for CRC-SCA). The EUROSCA and RISCA data are and CRC-SCA data will be available upon reasonable request via the Critical Path to Therapeutics for the Ataxias (CPTA, https://c-path.org/programs/cpta/). The data model used in SCAview can be found within the documentation page of SCAview and allow interoperability and reuse of data.

## Consortia EUROSCA study group

Heike Jacobi^5^, Sophie Tezenas du Montcel^11, 12^, Peter Bauer^13^, Paola Giunti^14^, Arron Cook^15^, Robyn Labrum^16^, Michael H Parkinson^17^, Alexandra Durr^11^, Alexis Brice^11^, Perrine Charles^18^, Cecilia Marelli^19^, Caterina Mariotti^20^, Lorenzo Nanetti^20^, Marta Panzeri^20^, Maria Rakowicz^21^, Anna Sulek^22^, Anna Sobanska^21^, Tanja Schmitz-Hübsch^23^, Ludger Schöls^24, 25^, Holger Hengel^24, 25^, Laszlo Baliko^26^, Bela Melegh^27^, Alessandro Filla^28^, Antonella Antenora^28^, Jon Infante^29, 30^, José Berciano^30^, Bart P. van de Warrenburg^31^, Dagmar Timmann^32^, Sandra Szymanski^33^, Sylvia Boesch^34^, Jun-Suk Kang^35^, Massimo Pandolfo^36^, Jörg B. Schulz^37, 38^, Sonia Molho^12^, Alhassane Diallo^39^, Thomas Klockgether^1,3^

### ESMI study group

Jennifer Faber^1, 3^, Marcus Grobe-Einsler^1, 3^, Demet Önder^1, 3^, Mafalda Raposo^40, 41^, João Vasconcelos^41, 42^, Manuela Lima^41^, Luís Pereira de Almeida^43, 44, 45^, Patrick Silva^43, 44, 45^, Inês Cunha^43^, Paola Giunti^14^, Hector Garcia-Moreno^14^, Katarina Manso^14^, Matthis Synofzik^24, 25^, Holger Hengel^24, 25^, Andreas Traschütz^24, 25^, Bart van de Warrenburg^31^, Judith van Gaalen^46^, Tessa Perbolt^31^, Khalaf Bushara^47^, Diane Hutter^47^, Heike Jacobi^5^, Jon Infante^29, 30^, Leire Manrique^29^, Andreas Thieme^32^, Friedrich Erdlenbruch^32^, Chiadi Onyike^48^, Ann Fishman^48^, Kathrin Reetz^37, 38^, Imis Dogan^37, 38^, Eva Ratai^49^, Jeremy Schmahmann^50, 51^, Magda Santana^43, 44, 52^, Jeannette Hübener-Schmid^53, 54^, Thomas Klockgether^1, 3^

### CRC-SCA study group

Tetsuo Ashizawa^7^, Karla P. Figueroa^55^, Susan L. Perlman^56^, Christopher M. Gomez^57^, George R. Wilmot^58^, Jeremy D Schmahmann^50, 51^, Sarah H Ying^59^, Theresa A Zesiewicz^60^, Henry L Paulson^61^, Vikram G Shakkottai^62^, Khalaf Bushara^47^, Sheng-Han Kuo^6^, Michael D Geschwind^63^, Guangbin Xia^64^, Stefan M. Pulst^65^, Sub H. Subramony^66^

### RISCA study group

Heike Jacobi^5^, Sophie Tezenas du Montcel^11, 12^, Sandro Romanzetti^37, 38^, Florian Harmuth^53^, Caterina Mariotti^20^, Lorenzo Nanetti^20^, Maria Rakowicz^22^, Grzegorz Makowicz^67^, Alexandra Durr^11^, Alessandro Filla^28^, Alessandro Roca^28^, Ludger Schöls^2, 25^, Holger Hengel^2, 25^, Jon Infante^29, 30^, Jun-Suk Kang^35^, Carlo Casalo^68^, Marcella Masciullo^69^, Laszlo Baliko^26^, Bela Melegh^27^, Wolfgang Nachbauer^70^, Katrin Bürk-Gergs^71, 72^, Jörg B Schulz^37, 38^, Olaf Riess^53^, Kathrin Reetz^37, 38^, Thomas Klockgether^1, 3^

### SCA Registry study group

Jennifer Faber^1, 3^, Marcus Grobe-Einsler^1, 3^, Demet Önder^1, 3^, Berkan Koyak^1, 3^, Kathrin Reetz^37, 38^, Thomas Klockgether ^1, 3^

11 Sorbonne Université, Paris Brain Institute, Inserm, INRIA, CNRS, APHP, 75013 Paris, France

12 Biostatistics Unit, Groupe Hospitalier Pitié-Salpêtrière, Paris, France

13 Institute of Medical Genetics and Applied Genomics, University of Tübingen, Tübingen, Germany

14 Ataxia Centre UCL Queen Square London United Kingdom

15 Department of Molecular Neuroscience, UCL, Institute of Neurology, London, United Kingdom

16 Neurogenetic Laboratory, National Hospital of Neurology and Neurosurgery, UCLH, London, United Kingdom

17 Department of Molecular Neuroscience, UCL, Institute of Neurology, London, United Kingdom

18 Hôpital de la Pitié-Salpêtrière, Département de Génétique, Paris, France

19 Service de Neurologie – CMRR, CHRU Gui de Chauliac, Mont-pellier, France

20 Genetics of Neurodegenerative and Metabolic Diseases, Fondazione-IRCCS Istituto Neurologico Carlo Besta, Milan, Italy

21 Department of Clinical Neurophysiology, Institute of Psychiatry and Neurology, Warsaw, Poland

22 Department of Genetics, Institute of Psychiatry and Neurology, Warsaw, Poland

23 Charité Universitätsmedizin Berlin, Klinik für Neurologie, Berlin, Germany

24 Department of Neurology and Hertie-Institute for Clinical Brain Research, University of Tübingen, Tübingen, Germany

25 German Center for Neurodegenerative Diseases (DZNE), Tübingen, Germany

26 Department of Neurology, Zala County Hospital, Hungary

27 Department of Medical Genetics, and Szentagothai Research Center, University of Pécs, Pécs, Hungary

28 Department of Neuroscience, and Reproductive and Odontos-tomatological Sciences, Federico II University Naples, Italy

29 Service of Neurology, University Hospital Marqués de Valdecilla (IDIVAL), University of Cantabria (UC) and Centro de Investigación Biomédica en Red de Enfermedades Neurodegenerativas (CIBERNED), Santander, Spain

30 Service of Neurology, University Hospital “Marqués de Valdecilla (IDIVAL)”, “Centro de Investigación Biomédica en Red de Enfermedades Neurodegenerativas (CIBERNED)”, University of Cantabria (UC), Santander, Spain

31 Department of Neurology, Donders Institute for Brain, Cognition, and Behaviour, Radboud University medical center, Nijmegen, The Netherlands

32 Department of Neurology and Center for Translational Neuroand Behavioral Sciences (C-TNBS), Essen University Hospital, University of Duisburg-Essen, Essen, Germany

33 Department of Neurology, St. Josef Hospital, University Hospital of Bochum, Bochum, Germany

34 Department of Neurology, Medical University, Innsbruck, Innsbruck Austria

35 Department of Neurology, University of Frankfurt, Frankfurt/M, Germany

36 Université Libre de Bruxelles (ULB), Neurology Service—ULB Hôpital Erasme, ULB Laboratory of Experimental Neurology, Brussels, Belgium

37 Department of Neurology, RWTH Aachen University, Pauwelsstraβe 30, 52074 Aachen, Germany

38 JARA—Translational Brain Medicine, Aachen-Jülich, INM 11, Germany

39 Sorbonne Universités, Université Pierre et Marie Curie (UPMC) Univ Paris, Institut Pierre Louis d’Epidémiologie et de Santé Publique, Paris, France

40 Instituto de Biologia Molecular e Celular (IBMC), Instituto de Investigação e Inovação em Saúde (i3S), Universidade do Porto, Porto, Portugal

41 Faculdade de Ciências e Tecnologia, Universidade dos Açores, Ponta Delgada, Portugal

42 Hospital Internacional dos Açores (HIA), Ponta Delgada, Portugal

43 Center for Neuroscience and Cell Biology, University of Coimbra, Coimbra, Portugal

44 Center for Innovation in Biomedicine and Biotechnology, University of Coimbra, Coimbra, Portugal

45 Faculty of Pharmacy, University of Coimbra, Coimbra, Portugal

46 Department of Neurology, Donders Institute for Brain, Cognition, and Behaviour, Radboud University medical center, Nijmegen, The Netherlands & Department of Neurology, Rijnstate Hospital, Arnhem, The Netherlands,

47 Department of Neurology, University of Minnesota, Minneapolis, Minnesota, USA

48 Johns Hopkins University School of Medicine, Baltimore, MD, U.S.

49 Athinoula A. Martinos Center for Biomedical Imaging, Department of Radiology, Massachusetts General Hospital, Boston, MA, USA

50 Ataxia Center, Cognitive Behavioral Neurology Unit, Laboratory for Neuroanatomy

51 Cerebellar Neurobiology, Department of Neurology Massachusetts General Hospital, Harvard Medical School, Boston, Massachusetts, USA

52 Institute for Interdisciplinary Research, University of Coimbra (IIIUC), Coimbra, Portugal

53 Institute of Medical Genetics and Applied Genomics, University of Tübingen, Tübingen, Germany

54 Centre for Rare Diseases, Medical Faculty, University of Tübingen, Tübingen, Germany

55 University of Utah, Salt Lake City, UT, USA

56 UCLA, Los Angeles, CA, USA

57 University of Chicago, Chicago, IL, USA

58 Emory University, Atlanta, GA, USA

59 Massachusetts Eye and Ear Infirmary

60 University of South Florida, FL, USA

61 University of Michigan, Ann Arbor, MI, USA

62 UTSW, Dallas, TX, USA

63 UCSF, San Francisco, CA, USA

64 Indiana University, Fort Wayne, IN, USA

65 University of Utah, Salt Lake City, UT, USA

66 University of Florida, Gainesville, FL, USA

67 Department of Neuroradiology, Institute of Psychiatry and Neurology, Warsaw, Poland

68 Department of Medico-Surgical Sciences and Biotechnologies “Sapienza” University of Rome, Rome, Italy

69 SPInal REhabilitation Lab (SPIRE), IRCCS Fondazione Santa Lucia, Rome, Italy

70 Department of Neurology, Medical University Innsbruck, Inns-bruck, Austria

71 Department of Neurology, Philipps University Marburg

72 Kliniken Schmieder Stuttgart-Gerlingen, Germany
