# Supplementary material for: SCAview: an Intuitive Visual Approach to the Integrative Analysis of Clinical Data in Spinocerebellar Ataxias
Source: Cerebellum. Author manuscript; Available in PMC 2024 Jun 1. (PMC10544694; doi:10.1007/s12311-023-01546-0)
Supplement: Supplementary1 [file NIHMS1916742-supplement-Supplementary1.pdf]

Supplementary Data

A common underlying data model was established. Here we show, examples for attribute mappings and transformation code mappings as well as calculations. While for some attributes a one-to-one mapping to a data model attribute ("Target_Attribute") was possible, for others transformations or calculations were necessary.

a)
Attribute Mapping

Source	Source_Attribute	Target_Attribute	Transformation
SCAregistry	sex	SEX	cm001
SCAregistry	yob	DOB	
SCAregistry	AOO	ONSET_GAIT	
CRC-SCA	DNATestingRepeatsExpandedAllel	LONG	
CRC-SCA	FunctionalStage	DISEASE_STAGE	cm012

b)
Code Mapping

Code_Mapping	Source_Value	Core_Equivalent
cm001	m	0
cm001	f	1
cm001	male	0
cm001	female	1
cm012	0	0
cm012	0,5	0
cm012	1	0
cm012	1,5	1
cm012	2	1
cm012	2,5	1
cm012	3	2
cm012	3,5	2
cm012	4	2
cm012	4,5	2
cm012	5	3
cm012	5,5	3
cm012	6	3

c)
Calculations

Source	Attribute	Function
CRC-SCA	ONSET_GAIT	ADDTIME(DOB, WALKINGPROBLEMSYEAR, unit=Y)
